# Contribution of the genomic and nutritional differentiation to the spatial distribution of bacterial colonies

**DOI:** 10.3389/fmicb.2022.948657

**Published:** 2022-08-23

**Authors:** Kenya Hitomi, Jieruiyi Weng, Bei-Wen Ying

**Affiliations:** School of Life and Environmental Sciences, University of Tsukuba, Tsukuba, Ibaraki, Japan

**Keywords:** spatial analysis, *Voronoi* diagram, genome reduction, colony size, single colony growth, agar plate, medium

## Abstract

Colony growth is a common phenomenon of structured populations dispersed in nature; nevertheless, studies on the spatial distribution of colonies are largely insufficient. Here, we performed a systematic survey to address the questions of whether and how the spatial distribution of colonies was influenced by the genome and environment. Six *Escherichia coli* strains carrying either the wild-type or reduced genomes and eight media of varied nutritional richness were used to evaluate the genomic and environmental impacts, respectively. The genome size and nutritional variation contributed to the mean size and total area but not the variation and shape of size distribution of the colonies formed within the identical space and of equivalent spatial density. The spatial analysis by means of the *Voronoi* diagram found that the *Voronoi* correlation remained nearly constant in common, in comparison to the *Voronoi* response decreasing in correlation to genome reduction and nutritional enrichment. Growth analysis at the single colony level revealed positive correlations of the relative growth rate to both the maximal colony size and the *Voronoi* area, regardless of the genomic and nutritional variety. This result indicated fast growth for the large space assigned and supported homeostasis in the *Voronoi* correlation. Taken together, the spatial distribution of colonies might benefit efficient clonal growth. Although the mechanisms remain unclear, the findings provide quantitative insights into the genomic and environmental contributions to the growth and distribution of spatially or geographically isolated populations.

## Introduction

Studies on colony growth are important for understanding microbial ecology (Ernebjerg and Kishony, 2012; Skandamis and Jeanson, 2015), as microorganisms grown on solid surfaces often form colonies (Jeanson et al., 2015). The growth environment plays an important role in microbial growth; for example, both nitrogen and carbon can cause catabolite repression (Simpson-Lavy and Kupiec, 2019; Nair and Sarma, 2021). Studies on the growth of single colonies observed that colony growth was dependent on the nutritional condition, agar concentration and presence of other cells (Harcombe et al., 2014; Warren et al., 2019; Díaz-Pascual et al., 2021). The colony growth pattern and dynamics could be perturbed by spatial antibiotics in the growth environment (Frost et al., 2018; Sharma and Wood, 2021), which might be caused by physical and mechanical interactions (Persat et al., 2015; Tronnolone et al., 2018). Despite many studies linking environmental conditions to colony growth patterns (van Gestel et al., 2014; Cole et al., 2015; Xiong et al., 2020), the spatial distribution of colony size has rarely been reported. The landmark study first applied spatial analysis of *Voronoi* diagram to observe the size variation of the colonies grown on identical plates (Chacón et al., 2018). The correlation of the colony size to the spatial area assigned to the colony (i.e., *Voronoi* area) was observed (Xue et al., 2021). Whether such a correlation in the spatial distribution of colonies was affected by the environmental conditions was required to be addressed. In addition to the environment, genomic information is another crucial factor for colony growth. Previous studies reported that mutations in *rpoS* inhibited colony maturation (Ito et al., 2008, 2009) and that *hemB* was responsible for the formation of small mutant colonies (Bates et al., 2003). In addition, small RNAs (sRNAs) were found to be involved in biofilm formation (Van Puyvelde et al., 2013; Bak et al., 2015; Parker et al., 2017). These studies investigated the relationship between genetic information and colony growth in terms of the contribution of specific genes or sRNAs. Besides these genetic changes of defined mechanism and/or function, the large deficiency of genomic fragments was supposed to disturb colony growth significantly. Both systematic single-gene knockout (Baba et al., 2006; Joyce et al., 2006) and genome reduction (Kato and Hashimoto, 2007; Mizoguchi et al., 2008; Karcagi et al., 2016) were intensively reported. The genome-reduced strains were successfully applied to studies on metabolic engineering (Lee et al., 2009), experimental evolution (Choe et al., 2019), growth prediction (Ashino et al., 2019), origin of life (Lu et al., 2022), and so on. Whether and how genome reduction contributes to growth dynamics have been intensively studied in liquid media (Karcagi et al., 2016; Kurokawa et al., 2016), which has led to valuable insights into adaptive evolution and niche expansion (Nishimura et al., 2017; Choe et al., 2019; Kurokawa et al., 2022). Nevertheless, the contribution of genome reduction to colony growth remains unclear, as the growth dynamics in liquid media are somehow different from those on solid surfaces (Tsuchiya et al., 2018). Consequently, whether the correlation in the spatial distribution of colonies was affected by the genome reduction was unclear.

Taken together, the present study employed the model bacterium *Escherichia coli* (*E. coli*) to address the questions raised above, that is, of how genome reduction and nutritional variation effected the spatial distribution of colonies and whether genomic and environmental contributions are common. An assortment of genome-reduced *E. coli* strains was used to evaluate the contribution of genomic gradient to colony spatial distribution. The environmental contribution to colony spatial distribution was evaluated upon nutritionally varied media, which were adjusted by the medium composition of either the concentration of glucose in the minimal medium or the ratio of the nutritional rich medium and the minimal medium.

## Results and discussion

### Parameters defined for colony growth and spatial distribution

**FIGURE 1:**
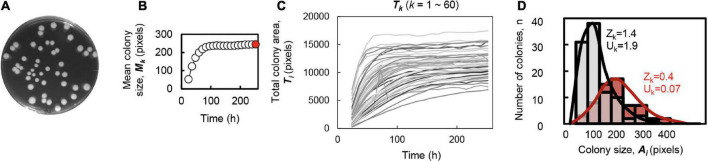
Parameters used for the colony spatial analyses. **(A)** Image of colonies on the agar plate. **(B)** Temporal changes in the averaged size of the colonies formed on a single plate. The steady state is indicated in red at the time point of 252 h, which is corresponded to the image shown in panel **(A)**. **(C)** Temporal changes in the total area of all colonies formed on a single plate. The gradation in gray indicates a total of 60 colonies located on the identical plate. **(D)** Histogram of the colony size within a single plate. As representative examples, both the histograms and the fitted distributions of the areas of the colonies grown on the plates are shown. Color variation (gray and red) represents two different plates. The calculated values of skewness (*Z*_*k*_) and kurtosis (*U*_*k*_) of the distributions are indicated.

A total of six *E. coli* strains of varied genome sizes, i.e., the wild-type W3110 strain (N0) and its derivates of reduced genomes (N3, N10, N14, N20, and N28) (Supplementary Figure 1A), were employed to evaluate the effect of genome size on colony growth and spatial distribution. The concentration gradient of either glucose in the minimal medium or Luria-Bertani (LB) in the mixed medium were applied to evaluate the effect of the nutritional variation on the colony growth and spatial distribution. Multiple dilution rates and replicates of the plating were conducted for each condition, and the temporal changes in colony growth were recorded for the following analyses (Supplementary Figure 1B). The statistical parameters related to colony growth and spatial distribution, which are summarized in Table 1, were evaluated as follows. To capture the spatial distribution of the colonies grown on each plate, the number (*D*_*k*_), the mean size (*M*_*k*_), the total area (*T*_*k*_), and the size variation (*V*_*k*_) of all colonies on the plate, as well as the skewness (*Z*_*k*_), and kurtosis (*U*_*k*_) of the distribution of colony size, which were introduced as the indicators of deviation from the normal distribution, were calculated (Figure 1 and Table 1). These parameters all described the size distribution of colonies located on the same solid space (agar plate).

**TABLE 1:** The parameters used.

*Single colony level*	
Colony	*C*_*i*_, *i* = 1∼n; n, number of colonies
Colony size	*A*_*i*_ (pixels), *i* = 1∼n; n, number of colonies
Voronoi area	*Vor*_*i*_ (pixels), *i* = 1∼n; n, number of colonies
Relative growth rate	*r*_*i*_ (h^–1^), *i* = 1∼n; n, number of colonies
Maximal size	*K*_*i*_ (pixels), *i* = 1∼n; n, number of colonies
***Spatial level***	

Agar plates	*P*_*k*_, *k* = 1∼n; n, number of plates
Spatial density	*D*_*k*_, *k* = 1∼n; n, number of plates
Mean colony size	*M*_*k*_ (pixels), *k* = 1∼n; n, number of plates
Total colony area	*T*_*k*_ (pixels), *k* = 1∼n; n, number of plates
Colony size variation	*V*_*k*_, *k* = 1∼n; n, number of plates
Skewness	*Z*_*k*_, *k* = 1∼n; n, number of plates
Kurtosis	*U*_*k*_, *k* = 1∼n; n, number of plates
*Voronoi* response	*Res*_*k*_, *k* = 1∼n; n, number of plates
*Voronoi* correlation	*Cor*_*k*_, *k* = 1∼n; n, number of plates

The parameters and abbreviations used in the study for describing the distribution and growth of colonies.

In addition, the growth dynamics at the single colony level were evaluated with two parameters: the relative growth rate (*r*_*i*_) and the steady size (*K*_*i*_). The spatial analysis according to the *Voronoi* diagram (Dale and Fortin, 2014; Baddeley et al., 2015) resulted in the parameters of the *Voronoi* area (*Vor*_*i*_) for individual colonies, the *Voronoi* response (*Res*_*k*_) and the *Voronoi* correlation (*Cor*_*k*_) for each plate (space). These parameters (Table 1) are further described in the corresponding results sections.

### Correlation between spatial density and size distribution of the colonies

**FIGURE 2:**
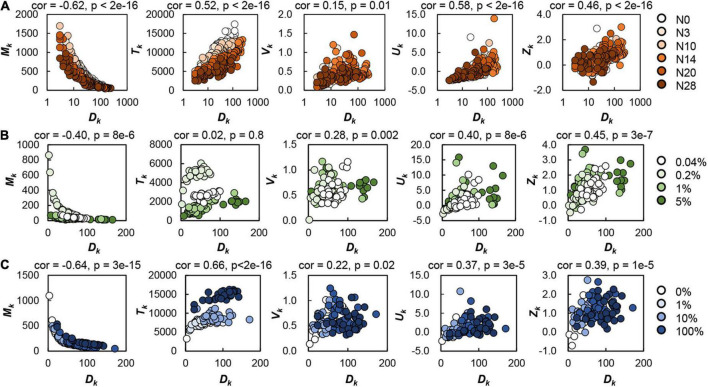
Effect of spatial density on colony formation. The number of colonies grown on the single plate (*D*_*k*_) was plotted against the calculated parameters relating to the colony size and distribution on the identical plate, i.e., the mean size (*M*_*k*_), total area (*T*_*k*_) and variation (*V*_*k*_) of the colonies, and the kurtosis (*U*_*k*_) and skewness (*Z*_*k*_) of the distribution of colony size. Circles represent the plates. Orange gradation (*N* = 284) indicates the genome size from the wild type to the reduced ones **(A)**. Green gradation (*N* = 117) represents the concentration gradient of glucose in the minimal medium M63 **(B)**. Blue gradation (*N* = 119) represents the ratio gradient of LB in the mixed medium M63LB **(C)**. Pearson’s correlation coefficients and the *p*-values are indicated.

The spatial density of the colonies determined the colony size and variation within the same space, independent of the genomic and nutritional variations (Figure 2). The number of colonies grown on the agar plate (*D*_*k*_) was significantly correlated with all five parameters representing the colony size and the size distribution (*M*_*t*_, *T*_*k*_, *V*_*k*_, *U*_*k*_, and *Z*_*k*_), regardless of genome reduction (Figure 2A), carbon abundance (Figure 2B), or nutritional richness (Figure 2C). As all these parameters described the colonies grown at steady state, the bias of the colony growth phase could be ignored. The common correlations demonstrated that the spatial distribution of colonies was largely influenced by the spatial density of colonies (i.e., the number of colonies per plate). Note that the colonies grown on the plates containing 0.2% glucose showed an enlarged *T*_*k*_, which might be because the medium composition (e.g., glucose concentration) was occasionally the optimized condition for bacterial growth (Millard et al., 2017; Ashino et al., 2019).

The increase in the spatial density of colonies led to a decrease in the mean size of colonies, whereas it was associated with an increase in the total area of colonies (Figure 2, left two panels). A larger number of small colonies might be more efficient in resource utilization than a smaller number of large colonies. The variation in colonies within the same space changed in response to the increased spatial density (Figure 2, middle panels). The higher density of the colonies on the plate (*D*_*k*_) resulted in the larger variation of colony size (*V*_*k*_), indicating the larger differentiation in growth of the colonies located on the same space. This was supported by the positive correlations of *D*_*k*_ to both *Z*_*k*_ and *U*_*k*_ (Figure 2, right two panels), as both the longer right tailed and the more sharped distributions, represented by larger *Z*_*k*_ and *U*_*k*_, respectively, indicated the biased growth of colonies.

### Genomic and nutritional differences correlated with colony size at the spatial level

**FIGURE 3:**
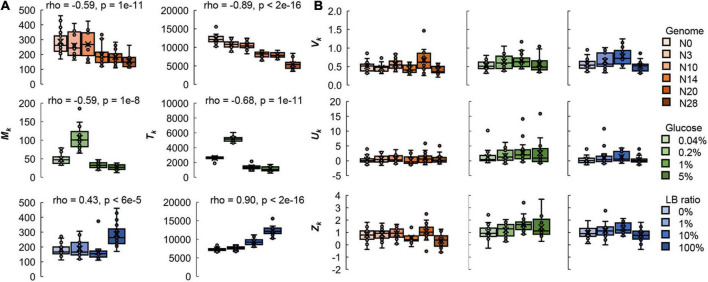
Boxplots of the global parameters relating to the size distribution of colonies. **(A)** Genomic and nutritional gradient-dependent parameters. The mean size (*M*_*k*_) and total area (*T*_*k*_) of the colonies on individual plates are shown. **(B)** Genomic and nutritional gradient independent parameters. The variation (*V*_*k*_) of the colonies and the kurtosis (*U*_*k*_) and skewness (*Z*_*k*_) of the distribution of colony size are shown. Tiny circles and crosses represent the parameters of individual plates of 25–75 colonies and their mean values, respectively. Gradations in orange, green, and blue indicate the genome size from the wild type to the reduced ones (*N* = 111), the concentration gradient of glucose in the minimal medium M63 (*N* = 78) and the ratio gradient of LB in the mixed medium M63LB (*N* = 80). The individual data points can be found in Supplementary Figure 3. Spearman’s correlation coefficients and the *p*-values, which were of statistical significance, are indicated.

Both the mean size and the total area of the colonies were significantly correlated with the genome size and nutritional variation; however, the variation and distribution of colony size remained constant (Figure 3 and Supplementary Figure 2). Note that only plates with a comparable number of colonies (25–75 colonies/plate) were used for the analyses to reduce the effect caused by the spatial density. As genome reduction was additively cumulative (Mizoguchi et al., 2008; Kurokawa et al., 2016), the negative correlation between colony size and the length of genomic deletion (Figure 3A) implied that it was the genome size but not the specific gene function that played a role in colony growth. The findings were consistent with the growth dynamics of genome-reduced *E. coli* strains in liquid media (Kurokawa et al., 2016; Nishimura et al., 2017).

The nutritional richness and the carbon abundance showed differentiated effects on colony growth. The mean size and total area of the colonies were negatively correlated with the concentration of glucose in the minimal medium, M63 (Figure 2B, left two panels), whereas they were positively correlated with the ratio of LB in the mixed medium, M63LB (Figure 2C, left two panels). The enrichment of the single resource of glucose and the overall nutritional richness (LB) in the media resulted in the reverse directional changes in the colony size. This result strongly suggested that the balance of the medium composition largely participated in colony growth, as similar as to population growth in liquid media (Ashino et al., 2019; Aida et al., 2022). In addition, the coefficient of variation of colony size (*V*_*k*_), kurtosis (*U*_*k*_), and skewness (*Z*_*k*_) of the size distribution of colonies all remained roughly comparable (*p* > 0.05), independent of either genome reduction or nutritional variation (Figure 3B). The results implied that the genetic and environmental variety disturbed the saturated colony size and/or the biomass abundance but not the size variation and distribution of colonies.

### Genomic and nutritional gradient-dependent *Voronoi* response and independent *Voronoi* correlation

**FIGURE 4:**
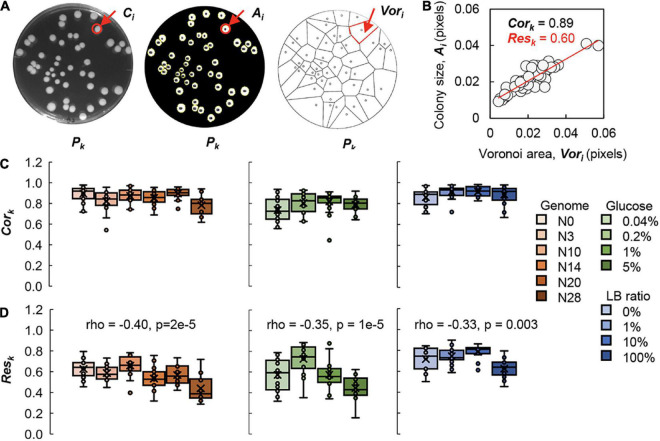
*Voronoi* diagram analysis. **(A)** Image analysis. The original image, the processed image and the *Voronoi* diagram of the agar plate (*P*_*k*_) are shown from left to right as an example. The single colony (*C*_*i*_), the corresponding colony size (*A*_*i*_) and its *Voronoi* area (*Vor*_*i*_) are indicated by arrows and highlighted in red. **(B)** Evaluation of *Voronoi* correlation and *Voronoi* response. The *Voronoi* correlation (*Cor*_*k*_) and *Voronoi* response (*Res*_*k*_) are defined as the correlation coefficient and the slope of linear regression of the relative *Voronoi* area and the relative colony size, respectively. As an example, the values of *Cor*_*k*_ and *Res*_*k*_ are indicated in black and red, respectively. Circles represent the colonies (*N* = 47). **(C)** Relationships of *Cor*_*k*_ to the genome size and nutritional gradient. Boxplots of *Cor*_*k*_ acquired from each plate are shown. **(D)** Correlations of *Res*_*k*_ to the genome size and nutritional gradient. Boxplots of *Res*_*k*_ acquired from each plate are shown. Tiny circles represent the *Cor*_*k*_ of individual plates of 25–75 colonies. The crosses indicate the average of *Cor*_*k*_. Gradations in orange, green and blue indicate the genome size from the wild type to the reduced ones (*N* = 111), the concentration gradient of glucose in the minimal medium M63 (*N* = 78) and the ratio gradient of LB in the mixed medium M63LB (*N* = 80). Spearman’s correlation coefficients and the *p*-values are indicated.

To evaluate the effect of the spatial distribution on the growth of colonies, the spatial analysis was performed using the *Voronoi* diagram, a practical method to divide the multiple distributed points into regions, as reported previously (Chacón et al., 2018; Xue et al., 2021). The parameter of the *Voronoi* area (*Vor*_*i*_) was calculated, indicating the space region on the plate assigned to the corresponding colony (Figure 4A). To discover the relationship between the relative size of the colony (*A*_*i*_) and the space in charge (*Vor*_*i*_), the correlation coefficient (*Cor*_*k*_) and the slope of linear regression (*Res*_*k*_) were evaluated (Figure 4B), as reported by previous studies (Chacón et al., 2018; Chacon et al., 2020). As *Cor*_*k*_ and *Res*_*k*_ were generally employed in the studies of spatial analysis for the patterns in geography and ecology (Dale and Fortin, 2014), the explanation in microbiology (e.g., pattern in colony growth) were the fairness of space division for colony growth and the efficiency of resource utilizing of the colony in the assigned space area, respectively.

The analytical results showed that *Cor*_*k*_ remained roughly constant, regardless of the changes in genome size or nutritional variety (Figure 4C). The value of *Cor*_*k*_ was close to 1, which revealed that the area charged by the colony was highly positively correlated with the growth capacity of the colony. The results clearly demonstrated the generality of the *Voronoi* correlation (*Cor*_*k*_) in colony growth, which strongly supported our previous finding (Xue et al., 2021) and indicated the commonly fair division of the space area for colony growth.

In comparison to the conserved *Voronoi* correlation, the *Voronoi* response was changed in response to the genomic and nutritional differences, which was complementary to the previous report on the wild-type bacterium growing on different carbon sources (Chacón et al., 2018). Weak but significant correlations of *Res*_*k*_ with both genome reduction and nutritional richness were detected (Figure 4D). The decrease in *Res*_*k*_ indicated that the decrease in colony size was correlated with the genome size and nutritional variation, although the assigned space for the colony, i.e., *Vor*_*i*_, remained equivalent. This strongly suggested the reduced efficiency of resource utilization associated with genome reduction and nutritional enrichment. Note that the findings were unbiased by the experimental (plating) conditions, as the *Vor*_*i*_ was independent of genome reduction or nutritional variation (Supplementary Figure 3). This might be the reason why the *Voronoi* correlation remained conserved. The spatial density of colonies (*D*_*k*_) was slightly influenced by either *Cor*_*k*_ or *Res*_*k*_ (Supplementary Figure 4); nevertheless, the conclusions were drawn from the plates of comparable *D*_*k*_.

### Genomic and nutritional differences correlated growth at the single colony level

**FIGURE 5:**
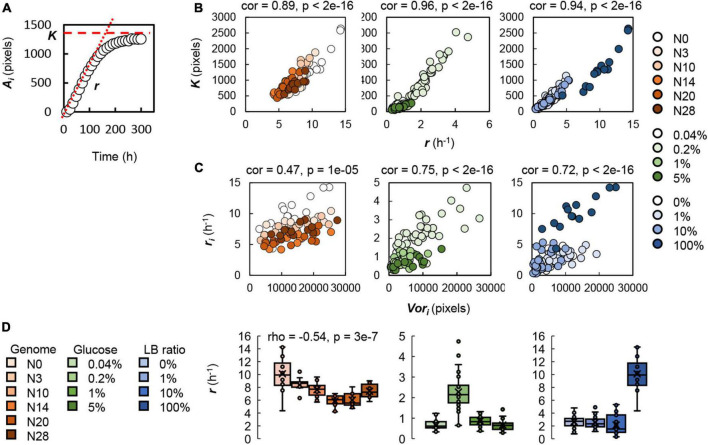
Single colony analysis. **(A)** Growth curve of a single colony. Circles indicate the temporal changes in the colony size. The relative growth rate (*r*) and the steady size of the colony (*K*) are indicated by red dashed lines. **(B)** Correlation between the relative growth rate and the steady size of the colonies. **(C)** Correlation between the *Voronoi* area and the relative growth rate. Circles represent the colonies. Gradations in orange, green and blue indicate the genome size from the wild type to the reduced ones (*N* = 80), the concentration gradient of glucose in the minimal medium M63 (*N* = 131) and the ratio gradient of LB in the mixed medium M63LB (*N* = 141). Pearson’s correlation coefficients and the statistical significance are indicated. **(D)** Relationships of genome reduction and nutritional richness with the growth rate. Boxplots of the relative growth rates acquired from each condition are shown. The tiny circles and the crosses represent the individual *r*_*i*_ and the average *r*_*i*_, respectively. Color variation in orange, green and blue and the numbers of single colonies used for the analysis are described above. Spearman’s correlation coefficients and the *p*-value, which was of statistical significance, are indicated.

To understand the genomic and nutritional differences correlated with changes in the spatial distribution, the growth of a single colony was analyzed, as a previous study proposed that the *Voronoi* response might be attributed to the growth rate (Chacon et al., 2020). According to the temporal changes in the size of a single colony, the relative growth rate (*r*) and the maximal colony size (*K*) were calculated (Figure 5A). Analyzing a total of 80 single colonies, we observed a significant positive correlation between the relative growth rate and the maximal size at the single colony level (Figure 5B). Although the colonies were grown on the various plates, the faster the colonies grew, the larger the colonies formed. It seemed to be a general mechanism, regardless of genome reduction or nutritional variety. Furthermore, the spatial analysis found a positive correlation between the growth rate and the *Voronoi* area at the single colony level (Figure 5C). This result indicated that the fast-growing colonies tended to have a large spatial area for colony growth in the future. Taken together, the increased growth rate was beneficial to occupying (assigning) the broadened space for continuous growth to achieve the enlarged size of the final population/colony. The coordinated relationship among *r*_*i*_, *K*_*i*_, and *Vor*_*i*_ at the single colony level well explained the universality of the *Voronoi* correlation at the spatial level (Figure 4C).

On the other hand, the relative growth rates of single colonies were correlated to genome reduction but not nutritional enrichment (Figure 5D). According to the coordination among *r*_*i*_, *K*_*i*_, and *Vor*_*i*_ (Figures 5B,C), the genomic gradient-correlated decrease in the growth rate triggered the reduced *Voronoi* area. Consequently, the *Voronoi* response declined in association with genome reduction, that is, the decreased *Vor*_*i*_ for the equivalent size of colonies carrying the reduced genomes. The contribution of genome reduction to both the colony size at the spatial level (Figure 3A, upper panels) and the *Voronoi* response in spatial distribution (Figure 4D, left panel) could be explained by the growth rate at the single colony level. However, the single colony growth analysis failed to explain the nutritional enrichment-associated decrease in the *Voronoi* response in spatial distribution. Although both genome reduction and the medium variety led to changes in colony growth, the mechanisms of the consequent changes in the *Voronoi* response seemed to be differentiated. Genetic restriction and resource limitation must have stimulated the varied regulatory pathways to achieve the maximal population size, although both caused growth decline.

### Hypothesis of fairness in spatial distribution for the efficiency of resource utilization

**FIGURE 6:**
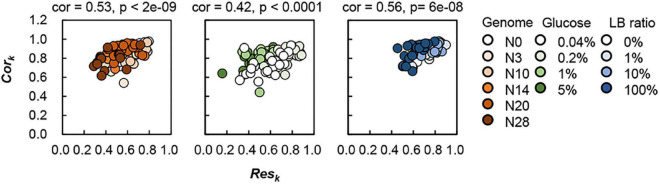
Relationship between the *Voronoi* response and *Voronoi* correlation. Circles represent the plates. Gradations in orange, green and blue indicate the genome size from the wild type to the reduced ones (*N* = 111), the concentration gradient of glucose in the minimal medium M63 (*N* = 78) and the ratio gradient of LB in the mixed medium M63LB (*N* = 80). Pearson’s correlation coefficients and the *p*-values are indicated.

It was an intriguing finding that the *Voronoi* correlation remained homeostatic, whereas the *Voronoi* response was differentiated in response to genetic and environmental changes. The steady *Cor*_*k*_ indicated that the colonies were grown in proportion to the assigned space (*Vor*_*i*_) regardless of the genomic and nutritional variety, which indicated that the resource space was distributed to the colonies according to their sizes. The fluctuation in *Res*_*k*_ indicated that the magnitudes of the changes in colony size of the identical *Vor*_*i*_ were changed due to genomic and nutritional interruptions, which indicated that the efficiency of resource utilization was different depending on the genome and environment. Although *Cor*_*k*_ remained approximately steady, its slight change was somehow linked to the large fluctuation in *Res*_*k*_. A weak but significant correlation between *Cor*_*k*_ and *Res*_*k*_ was observed (Figure 6). The spatial distribution of colonies was supposed to benefit the efficient utilization of space and/or nutritional resources. This finding well agreed with the natural relationship that the larger allocated region allowed more resources available for population increase. This relationship seemed to be true not only for the homogeneously growing populations in liquid media but also for the geographically isolated populations. The results quantitatively proved the ecological rule of the colony growth in response to its spatial distribution, which was likely common in *E. coli* independent of the genomic and nutritional variation.

The genetic and environmental dependent changes in *Res*_*k*_ were supposed to be decided by the colony growth rate. As a consequence of ecological evolution (Sexton et al., 2017), the growth rate was supposed to be negatively correlated with the population size (Luckinbill, 1978; Engen et al., 2013), which is known as the trade-off mechanism (Cavalier-Smith, 1980; Weisse et al., 2015; Ferenci, 2016). However, instead of a trade-off, a positive correlation was observed in colony growth (Figure 5B). The decreased growth rate resulted in a small colony, which was assigned to the identical *Vor*_*i*_. Consequently, the *Voronoi* response, the slope between colony size and *Vor*_*i*_, decreased. The disappearance of the trade-off between colony growth rate and maximal colony size well explained the changes in the *Voronoi* response. As the trade-off mechanism was explained by the metabolic and enzymatic costs (Molenaar et al., 2009; Noor et al., 2014; Wortel et al., 2018), whether the positive correlation between growth rate and growth max was caused by any other alternative mechanism involved in metabolic and/or cellular pathways remains to be addressed in the future.

The systematic survey on colony growth evaluated the impacts of genome reduction and nutritional richness on the bacterial spatial distribution. The genetic and environmental dependent *Voronoi* response associated with the homeostatic *Voronoi* correlation was observed. The genome reduction-mediated growth decrease, which was observed in the uniformed populations grown in the liquid media (Kurokawa et al., 2016), was demonstrated to be general in the spatially separated colonies grown on the solid media. It seemed that it was the abundance but not the specificity of the genetic information that participated in the bacterial population propagation. In summary, the present study is the first to connect genomic and nutritional variety with the spatial distribution of bacterial colonies. These findings provide quantitative insights into the genomic and environmental contributions to the growth and distribution of spatially or geographically isolated populations. Since only an assortment of the laboratory engineered *E. coli* strains grown in the well-controlled conditions were examined in the present study, further investigation of different microbes is required to draw a universal conclusion on the fairness in the spatial distribution of colonies for efficient clonal growth. The spatial patterns and growth of colonies were supposed to be affected by multiple factors, such as the cross-sectional structure of colonies, the cellular interaction within a single colony, the interaction between neighboring colonies, and the nutritional diffusion in agar media. Clarifying these underground mechanisms is highly intriguing to connect bacterial morphology to colony life.

## Materials and methods

### *Escherichia coli* strains

A total of six *E. coli* strains were used, including the wild-type *E. coli* strain K-12 W3110 and five of its derivative genome-reduced strains. These six strains were designated Nos. 0, 3, 10, 14, 20, and 28 (Supplementary Figure 1A), as described previously (Kurokawa et al., 2016; Nishimura et al., 2017). The lengths of deleted sequences are 88.7, 481.4, 709.5, 899.0, and 982.4 kb for strains Nos. 3, 10, 14, 20, and 28, respectively. The *E. coli* strains were from the KHK collection (Mizoguchi et al., 2008), distributed by the National BioResource Project, National Institute of Genetics, Shizuoka, Japan.

### Media and culture

Both the rich medium LB (Sigma) and the minimal medium M63, of which the chemical composition was described in detail previously (Kurokawa and Ying, 2017), were used. Nutritional variation of the media was prepared either by diluting LB with M63 or by varying the amount of glucose in M63, which resulted in four different nutritional levels of 100, 10, 1, and 0% LB media and 5, 1, 0.2, and 0.04% glucose M63 media, respectively. The glycerol stocks of the *E. coli* strains, prepared beforehand as previously described (Kurokawa et al., 2016; Kurokawa and Ying, 2017), were diluted to a final concentration of ∼1,000 cells/ml. The diluted culture solutions were further diluted twofold, which led to different initial cell concentrations for plating (Supplementary Figure 1B). The media used for the dilution was equivalent to the media used for the agar plate for colony growth. For colony formation, every 100 μl of the diluted bacterial solution was plated on each plate (1.5% agar). The plates were incubated at 37°C in an incubator (THS030PA, ADVANTEC). More than 10 replicates were conducted for each dilution rate of each strain at each medium condition. Approximately 600 plates were inoculated and incubated for colony growth.

### Imaging

The plates were photographed with a high-sensitivity monochrome CCD camera of a gel imager (AE-6932GXES print graph, ATTO Co., Ltd.). Brightness, contrast, saturation, hue, sharpness, OSD time, and exposure time were set as previously described (Xue et al., 2021). Temporal changes in colony growth were captured at intervals of 12 h (Supplementary Figure 1B). The imaging of the plates (colony growth) was started at 24 h after inoculation and stopped at 300 h. The steady state of the colonies was determined at 252 h. The images were saved as TIF files and subjected to the computational analysis described in the following sections. A total of approximately 5,000 images of the temporal changes in colony growth were acquired from 520 plates, which were qualified for imaging and computational analysis.

### Colony analysis and parameter calculation

Image data analysis was performed with Fiji, an open-source image processing package based on ImageJ.^1^ Background subtraction and binarization were performed on the acquired image data, and the edges of the plates were removed as noise. Only the images of high-resolution colonies were processed for the analysis. The coordinates of the center of each plate, the location of the colonies and the corresponding colony size *A*_*i*_ (*i* = 1∼n; n, number of colonies in the plate) were automatically calculated by the command Fiji. The number of colonies in each plate was defined as the colony density, *D*_*k*_ (*k* = 1∼N; N, number of plates). According to the calculated *A_*i*_*, the total area of colonies in each plate *T*_*k*_, the average colony size *M*_*k*_, and the colony size variation *V*_*k*_ were calculated. Skewness (*Z*_*k*_) and kurtosis (*U*_*k*_) were calculated using the “kurtosis” and “skewness” functions in the e1071 package of the R programming software. The calculations of these parameters were performed according to the following equations (Eqs. 1–6). These parameters are summarized in Table 1 and are shown in Figure 1.


(1) $$T_{k}=\sum_{i=1}^{n}A_{i}$$

(2) $$M_{k}=\frac{\sum_{i=1}^{n}A_{i}}{D_{k}}$$

(3) $$s\sqrt{\frac{\sum_{1}^{n}\left(A_{i}-M_{k}\right)}{n-1}}$$

(4) $$m_{r}\,\sum_{i}\frac{{\left(A_{i}-M_{k}\right)}^{r}}{n}$$

(5) $$V_{k}=\frac{s}{M_{k}}$$

(6) $$Z_{k}={\text{m}}^{3}/{\text{s}}^{3}$$

(7) $$U_{k}=m^{4}/s^{4}-3$$

### *Voronoi* diagram analysis

The *Voronoi* region, *Vor*_*i*_ (i = 1∼n; n, number of colonies), which is the theoretical region assigned to each colony, was calculated according to the following formula, as previously described (Xue et al., 2021).


(8) $$C=\left\{c_{1},c_{2},\ldots ,c_{n}\right\}$$

(9) $$V\,\left(c_{l}\right)=\left\{c|d\,\left(c,c_{l}\right)\leq d\,\left(c,c_{m}\right),m\neq l\right\}$$

(10) $$d\left(c,cm\right)\sqrt{{\left(x-x_{m}\right)}^{2}+{\left(y-y_{m}\right)}^{2}}$$

(11) $$d\left(c,cl\right)\sqrt{{\left(x-x_{l}\right)}^{2}+{\left(y-y_{l}\right)}^{2}}$$

Here, *V(c_l_)* and *d* represent the *Voronoi* area of the colony c_*l*_ and the function of the distance, respectively. l and m are natural numbers less than or equal to *i*, the number of colonies. x and y represent the X and Y coordinates of the colonies on the plates, which were used for the *Voronoi* division. The colonies and the agar plate were considered the genetic points (*c_1_, c_2_, …, c*_*n*_) and the metric space (*C*), respectively. The tessellation {*V(c_1_), V(c_2_), …, V(c_*k*_*)} refers to the *Voronoi* diagram. The *Voronoi* areas were calculated with the function “dirichlet” in the spatial statistics package “spatstat” in the software R.

### Single colony growth analysis

The growth curves of a total of 80 colonies were analyzed in detail. To avoid the bias caused by the colony density, plates with an equivalent number of colonies (5–11 colonies/plate) were subjected to growth analysis. The growth rate of a single colony at a certain time point, *t*, was defined as the slope of two neighboring records at time points *t* and *t-12* (Eq. 10.1).


(12) $${\text{r}}_{\text{t}}\left(h^{-1}\right)=\frac{A_{\left(t\right)}-A_{\left(t-12\right)}}{12}$$

Here, *r*_*t*_ and *A*_(t)_ represent the growth rate and the colony size at time point *t*, respectively. To avoid outliers, the maximal slope, *r*_*t*_, was averaged with its two neighboring slopes, *r*_(*t*–12)_ and *r*_(*t*+12)_. The advantages and methodology were described previously (Tsuchiya et al., 2018; Ashino et al., 2019). The resultant mean value was determined as the relative growth rate of colony *i* (Eq. 10.2).


(13) $${\text{r}}_{\text{i}}\left(h^{-1}\right)=\frac{r_{\left(t-12\right)}+r_{t}+r_{\left(t+12\right)}}{3}$$

## Data availability statement

The raw data supporting the conclusions of this article will be made available by the authors, without undue reservation.

## Author contributions

B-WY conceived the research. KH and JW conducted the experiments. KH and B-WY analyzed the data and wrote the manuscript. All authors contributed to the article and approved the submitted version.

## Footnotes

1
https://imagej.nih.gov/ij/
