# Supplementary material for: Contribution of the genomic and nutritional differentiation to the spatial distribution of bacterial colonies
Source: Front Microbiol. 2022 Aug 23;13:948657. doi: 10.3389/fmicb.2022.948657 (PMC9448356; doi:10.3389/fmicb.2022.948657)
Supplement: Supplementary file 1 [file Presentation_1.pdf]

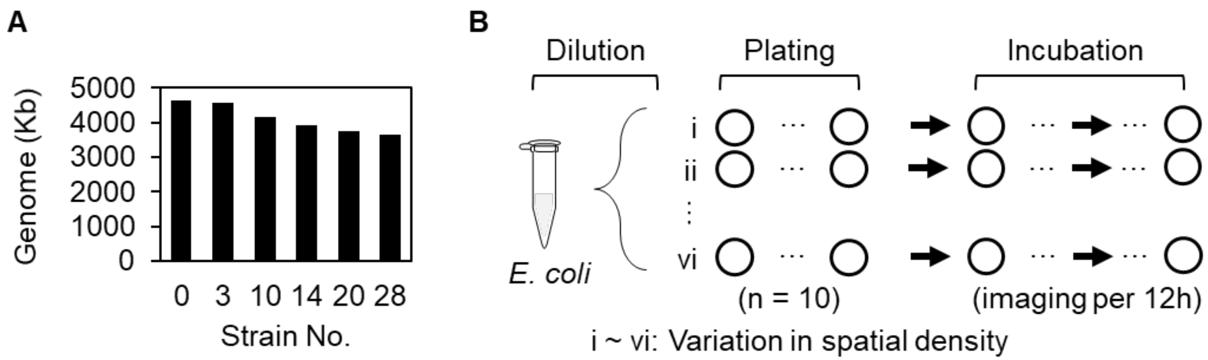

Figure S1 Experimental design. The genome sizes of the *E. coli* strains used in the study are shown (**A**). The lengths of deleted sequences are 88.7, 481.4, 709.5, 899.0, and 982.4 kb for strains Nos. 3, 10, 14, 20, and 28, respectively. The experimental procedures of culture dilution, plating, incubation, and imaging are illustrated (**B**). Multiple dilution rates and replicates for each condition are indicated.

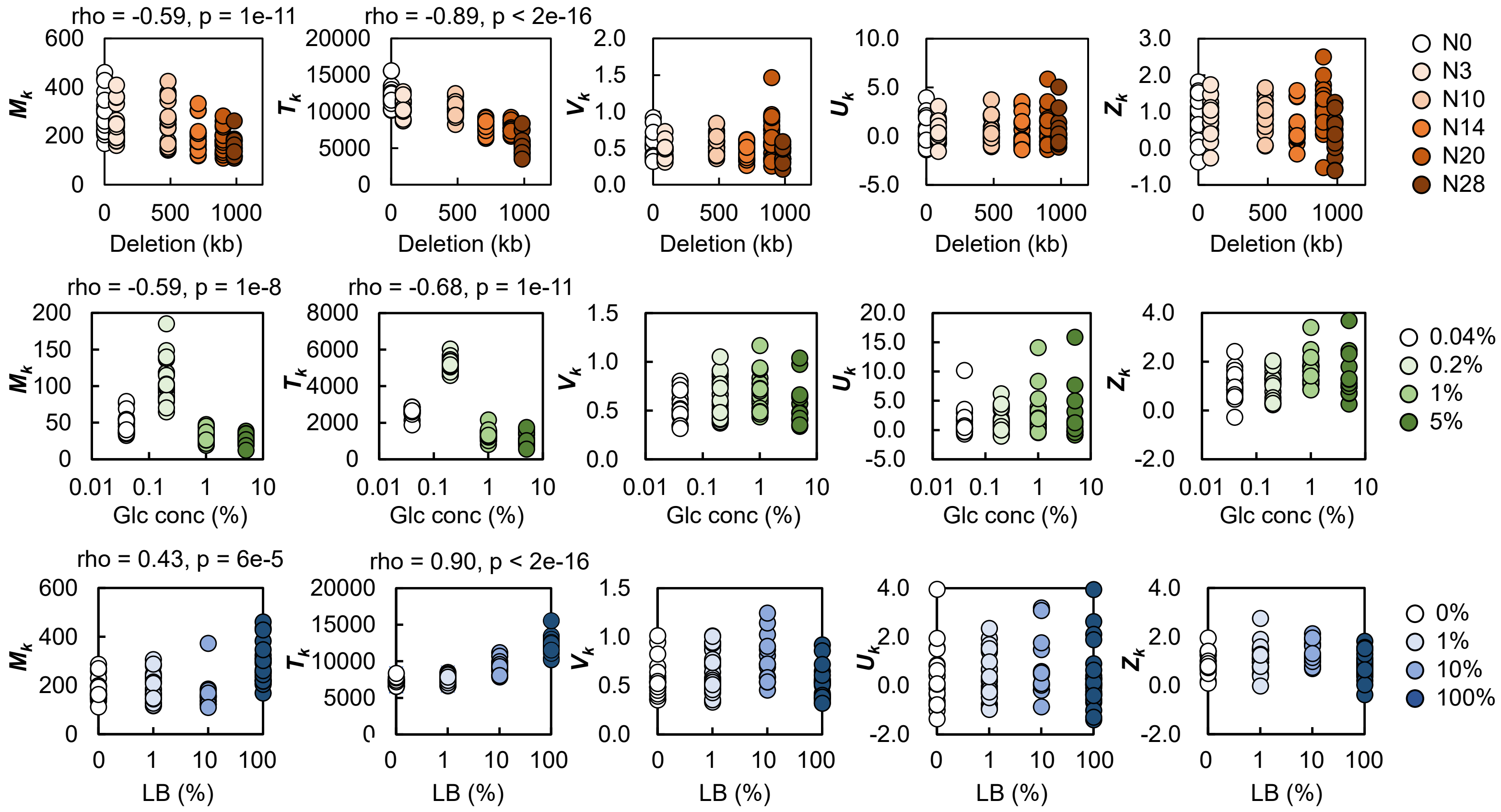

Figure S2 Effect of the genomic and nutritional differences on colony formation. The reduced size of the genome and the nutritional richness are plotted against the parameters relating to the colony size and distribution on the identical plate, i.e., the mean size (M_k), total area (T_k) and variation (V_k) of the colonies, and the kurtosis (U_k) and skewness (Z_k) of the distribution of colony size. Circles represent the plates. The corresponding boxplots are shown in Fig. 3. Gradations in orange, green and blue indicate the genome size from the wild type to the reduced ones (N=111), the concentration gradient of glucose in the minimal medium M63 (N=78) and the ratio gradient of LB in the mixed medium M63LB (N=80). Spearman's correlation coefficients and the p values, which were of statistical significance, are indicated.

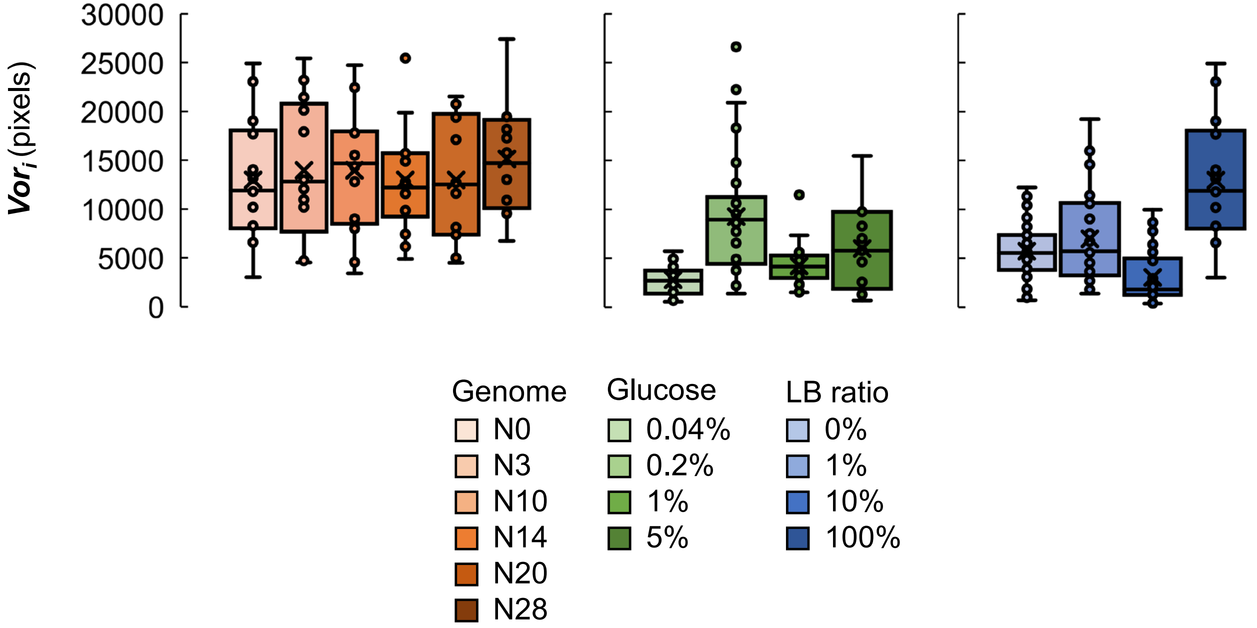

Figure S3 Effect of the genomic and nutritional differences on the *Voronoi* area. Boxplots of the *Voronoi* areas assigned for individual colonies are shown. The tiny circles and the crosses represent the individual Vor_i and the average of Vor_i, respectively. Gradations in orange, green and blue indicate the genome size from the wild type to the reduced ones (N=80), the concentration gradient of glucose in the minimal medium M63 (N=131) and the ratio gradient of LB in the mixed medium M63LB (N=141).

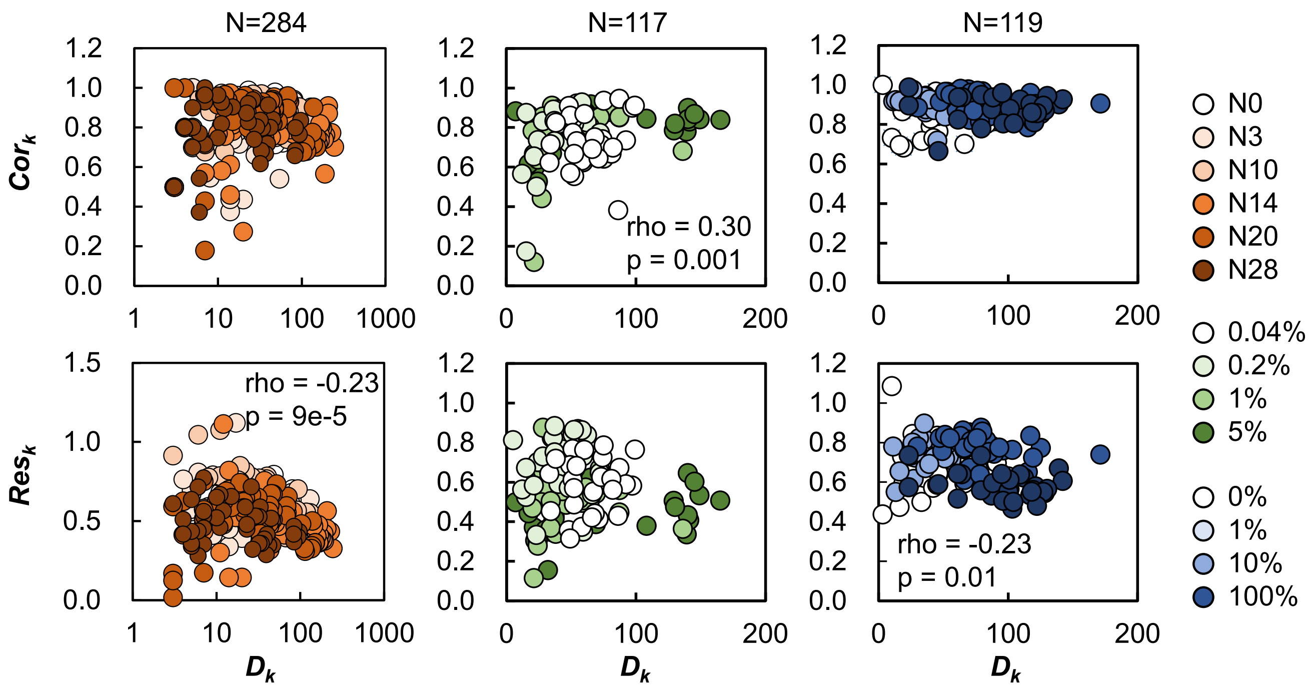

Figure S4 Effect of the spatial density on the *Voronoi* correlation and *Voronoi* response. The upper and bottom panels represent the relationships of the spatial density (D_k) to the *Voronoi* correlation and the *Voronoi* response, respectively. Circles represent the plates, and the numbers of plates used are indicated. Circles represent the colonies. Gradations in orange, green and blue indicate the genome size from the wild type to the reduced ones, the concentration gradient of glucose in the minimal medium M63 and the ratio gradient of LB in the mixed medium M63LB. Spearman's correlation coefficients and the *p* values, which were of statistical significance, are indicated.
